# Supplementary material for: Transition to a virtual model of physiotherapy and exercise physiology in response to COVID-19 for people in a rural Australia: Is it a viable solution to increase access to allied health for rural populations?
Source: PLoS One. 2023 Jan 20;18(1):e0280876. doi: 10.1371/journal.pone.0280876 (PMC9858084; doi:10.1371/journal.pone.0280876)
Supplement: S1 File — Patient interview and clinician focus group interview guides. (DOCX) [file pone.0280876.s001.docx]

**Supplementary material 1. Patient Interviews and clinician focus group interview guides.**

**SEMI STRUCTURED INTERVIEWS WITH PATIENTS**

1. **Background info**
2. What problem were you seeking treatment for?
3. **How did you find the X 2U virtual program?**

Probes:

- 1. Have you tried virtual healthcare before? If yes, in what context / with what health professional/s?
  2. Have you had regular (ie, in person) physio and/or EP before?
  3. Satisfaction with the virtual program
  4. Convenience (eg, no travel)
  5. Challenges (eg, lack of hands-on assessment / therapy)
  6. Clinician’s ability to diagnose and treat your problem

1. **What forms of remote support were you provided with?**

Probes:

- 1. Physitrack app, email, text message, phone calls, video conferencing

1. **What things did you find most helpful in the virtual consultation?**

Probes:

- 1. Physitrack app, paper version of exercise program
  2. Remote support provided by clinician

1. **What things did you find least helpful in the virtual consultation?**

Probes:

- 1. Physitrack app, paper version of exercise program
  2. Remote support provided by clinician
  3. Would you consider using telehealth again for physio / EP problems?

**FOCUS GROUP INTERVIEWS WITH CLINICIANS**

1. **What was your experience of implementing the virtual X2U service?**

Probes:

- 1. What worked well?
  2. What were challenges?
  3. What aspects you would change / not change?
  4. Experience of conducting a virtual assessment vs in person?
  5. Experience of providing remote support for therapy vs in person?
  6. Client response to the virtual service?
  7. Did you require more/less/same time for each patient consult vs in person?
  8. Were there some conditions where this approach worked particularly well or not so well? If so why?

1. **Skills and supports to implement a virtual service?**

Probes:

- 1. How prepared did you feel you were to transition to providing virtual healthcare?
  2. Did you receive any training/skills development to provide virtual healthcare? If yes, please describe
  3. How did you adapt your clinical approach to deliver services virtually?
  4. How much administrative support was required, compared to usual in person consults?
