# Supplementary material for: Transition to a virtual model of physiotherapy and exercise physiology in response to COVID-19 for people in a rural Australia: Is it a viable solution to increase access to allied health for rural populations?
Source: PLoS One. 2023 Jan 20;18(1):e0280876. doi: 10.1371/journal.pone.0280876 (PMC9858084; doi:10.1371/journal.pone.0280876)
Supplement: Attachment — Submitted filename: response to reviewers.docx [file pone.0280876.s005.docx]

**Journal Required Revisions**

Please review your reference list to ensure that it is complete and correct. If you have cited papers that have been retracted, please include the rationale for doing so in the manuscript text, or remove these references and replace them with relevant current references. Any changes to the reference list should be mentioned in the rebuttal letter that accompanies your revised manuscript. If you need to cite a retracted article, indicate the article’s retracted status in the References list and also include a citation and full reference for the retraction notice.

*We have reviewed our reference list and ensured that it is complete and correctly formatted. None of the references we have cited have been retracted. We have added three new references (references #29, #33 and #34) to the revised manuscript to address reviewer comments, as detailed below.*

**Review Comments to the Author**

**Reviewer #1:** Thank you for your thought-out responses to the comments raised. I appreciate your work in this area and the foundation from which future work can consider equitable access to virtual care in rural populations.

*Thank you for the overall positive comment.*

**Reviewer #3:** General comments:

Thank you for this interesting themed article. This concept is important to capture data and provide evidence to support continued use of telehealth/virtual care, particularly in the rural setting to increase access to services. I would like to encourage the authors to continue research in this field following on with planned studies targeting improving virtual care services. Meaningful results can be useful in lobbying government for continued funding for services delivered virtually.

*Thank you for the overall positive comment.*

It is understandable this study was unplanned and reactive to the unexpected scenario of the lockdown and does have some limitations in regards to small sample size etc but provides useful themes and a base for further research. It could also be noted that some of the initial patient/clinician concerns of moving to a virtual model of care may now be less of a worry today as society has become more accepting and experienced with this mode of communication since the lockdowns.

*Thank you, this is true. However, while society has become more experienced with virtual communication, virtual provision of care is not always feasible, preferred or delivered well, as indicated by our results. Providing patients with the choice of in-person or virtual care is important, particularly for rural patients where access barriers are known issues. We have added this point to the Discussion (lines 491-492):*

*“Ultimately, offering patients choice in how they access care may improve patient outcomes and treatment adherence (29).”*

It would be useful to include a definition of virtual healthcare in your introduction to highlight it is healthcare of a real patient delivered virtually/via telehealth (not a study examining virtual patients ie. Simulated patients for clinical training)

*We have added a definition of virtual healthcare to the Introduction (lines 53-54):*

*“Virtual healthcare, ie, healthcare delivered via telephone or video, has the potential to improve access to allied health services for people living in rural areas (8).”*

When considering the feasibility of virtual care and the methods use include video and/or phone, this adds a level of complexity to analysis. Although easiest to provide care in person, care and exercise instruction via video has some challenges, but care via phone is very difficult as the element of instruction or demonstration is removed. The authors might like to add clarity around proportion of phone vs video consultations, and if phone was only used for interviewing not conducting an exercise session with the patient or if this influenced clinician confidence.

*Thank you for highlighting this issue. We have included in the Results the proportions of video and phone consultations over the study period (lines 275-276), both of which were highly utilised:*

*“Clinicians delivered virtual healthcare primarily through video and/or phone (93% and 85%, respectively, across the study period); the mode was determined by the technology available to the patient.”*

*From the dataset we were unable to ascertain which type of virtual consultation was used for assessment versus intervention, or whether the type of consultation influenced clinician confidence. From some of the raw qualitative data we ascertained that the type of virtual consultation provided was determined by patient preference, which were influenced by logistics (eg, lines 335-340), patient familiarity with technology and internet connectivity. We have addressed these points in the Conclusion (lines 512-517):*

“*However, care needs to be taken to identify patients who would benefit most from such models, as high uptake of virtual healthcare in rural areas is not always guaranteed (22). Certain populations, eg, people with complex balance and/or cognitive impairment (11, 13) and people unfamiliar with technology (25, 32), appear not to benefit from virtually delivered exercise interventions. Poor internet and/or mobile phone connectivity in more remote areas may further impact uptake of virtual healthcare (13, 33).*”

In your discussion, you might like to consider either via supplementary material or additional reference(s), how the concerns identified in the each of the themes could be addressed to improve the virtual experience for the patient/clinician. Eg theme: “adaptation of program element”. Have the governing bodies or clinic developed any guidelines for the specific safety considerations to be made when using virtual care? Are there any other guidelines or protocols that have since been developed that link with the themes or do your themes lead you to suggest guidelines that could/should be developed that would be of use to other clinicians?

*Thank you for the suggestion to improve the experience of virtually delivered healthcare for both patients and clinicians, we have added these clinical implications to the Discussion. Specifically, we have outlined how some of the concerns identified in the themes of patient complexity (lines 450-456) and clinician flexibility (lines 497-501) should be addressed. In the Conclusion (lines 521-523) we have also referenced a recent clinical practice guideline (reference #34) published to assist clinicians embed virtual care into routine clinical practice; this guideline reinforced some of our findings.*

*These are the corresponding changes to the manuscript, listed in the order noted above:*

*“This was reflected in our qualitative findings where clinicians and patients alike felt that those with more complex conditions received more intensive therapy in-person. Clinically, it appears that triaging patients to offer virtual care to those with less complex needs and treating those with more complex needs in person may improve patient outcomes.”*

*“Similar to prior research, clinician confidence was higher among those who developed and used their skillset in delivering virtual healthcare on a regular and ongoing basis (30). This suggests the need for clinicians who will provide care virtually to maintain a regular caseload of such patients, rather than providing virtual care on an ad hoc basis. Interestingly, we and others found that clinical competence in successfully delivering healthcare virtually required both professional expertise (30) and a willingness to be flexible in their approach to work (31).”*

*“our results highlight the importance of establishing a therapeutic relationship and ensuring people have access to adequate resources, including access to and familiarity with technology as well as access to appropriate exercise equipment. Our findings are consistent with and reinforced in recently published guidelines for embedding virtual care into clinical practice (34).”*

Specific changes:
Line 57-65: Consider making minor rewording changes to this paragraph to clarify that this style of care was being used in rural/remote settings (and even city settings due to the lockdown) during the pandemic, not just the one clinic that was being studied.

*We have reworded this paragraph to clarify that virtual delivery of healthcare was used during Covid lockdowns across rural and metropolitan settings (lines 74-79):*

*“The COVID-19 pandemic required providers in both rural and metropolitan settings to offer patients their physiotherapy and exercise physiology services through a new service delivery model using only virtual health. For some providers, including the rural allied health provider studied herein, patients who lived close to the clinic and preferred to use the traditional in-person service during the pandemic could opt to do so.”*

Line 324: Theme titled “things occurring during treatment”, perhaps reconsider to avoid using “things” eg. Occurrences during virtual treatment

*We have reworded this theme to “conduct of virtual treatment” throughout the manuscript (lines 39-41, 298 and 324).*

Thank you for considering the rural/remote population and investigating how to increase access to healthcare in both pandemic and non-pandemic settings.

*Thank you for the overall positive comment.*
