# Supplementary material for: Transition to a virtual model of physiotherapy and exercise physiology in response to COVID-19 for people in a rural Australia: Is it a viable solution to increase access to allied health for rural populations?
Source: PLoS One. 2023 Jan 20;18(1):e0280876. doi: 10.1371/journal.pone.0280876 (PMC9858084; doi:10.1371/journal.pone.0280876)
Supplement: Attachment — Submitted filename: Reviewer Comments.docx [file pone.0280876.s003.docx]

Thank you for allowing me to review this important and timely work. Below are suggested major revisions to increase the clarity and impact of this work.

**ABSTRACT**

1. In Line 5 can you clarify that this is a rural practice consisting of physiotherapy and exercise physiology providers? As worded, it appears there are only 2 providers at the practice.
2. Line 6, please state how was the virtual model of care delivered (e.g., phone, video, any integration with apps)?
3. Please clarify whether exercise physiologists are considered clinicians in Australia and adjust the wording throughout the manuscript accordingly.
4. Line 9, please state the kind of mixed-methods approach utilized. It appears to be convergent but will need clarity.
5. Line 9-13 is a lengthy sentence and a needs restructuring for clarity (e.g., as written the monthly surveys are extracted from the database, is that correct?)
6. Line 20-21, it may not be appropriate to directly compare satisfaction of in-person to virtual to patients who may not have experienced both. This comment extends throughout the manuscript. My thought is that this data is descriptive.
7. Line 25-26, the themes as outlined do not make sense as standalones without context (e.g., appropriateness of the program, things occurring during treatment, communication). Please add more clarity and description.

**INTRODUCTION**

8. Line 51, can you clarify which conditions? It would be helpful to know if this was a controlled factor in the historical controls.
9. Lines 64-65, please re-word as this study did not directly measure access and equity.

**METHODS**

10. Line 72, please state the kind of mixed methods approached utilized.
11. Lines 82-83 appear missed place in the first paragraph briefly outlining the design.
12. Please add a section outlining the program delivery (mode, platform, length of sessions, rough structure of sessions, technology support/infrastructure for troubleshooting)
13. Please add the length of the interviews/focus groups and conceptual model guiding the interview questions (or more detail on how you developed interview/focus questions and probes). It may be helpful to add the interview guide to the supplementary/appendix material.
14. Please clarify whether the two focus groups were separated by discipline of mixed.
15. Lines 129-136 would be more appropriate in the first paragraph of the methods section.
16. Line 135, from the description of the clinical survey it did not appear that any questions were directed at “readiness” but more confidence/self-efficacy. Please clarify.
17. Were demographic data and characteristics collected on the providers? May give context when triangulating quant and qual data.
18. Line 142-143, it may be appropriate to disclose in the results how many patient satisfaction scores were reported as “no change” in the final data.
19. Lines 143-145, per my comment previously, it may not be appropriate to directly compare satisfaction of in-person to virtual to patients who may not have experienced both.
20. Line 155 please include mention of the de-brief in the methods section and not analysis.
21. Please provide additional detail on whether a codebook was created, how many researchers participated, how many discussions to reach a consensus. Did you complete any form of member checking to ensure accuracy of the interpretations? It may be helpful to add the codebook to the supplementary/appendix material.
22. The data analysis section needs a section on the mixed methods analysis and how you triangulated the data.

**RESULTS**

23. Please restructure the results staring with a section on “Patient Characteristics” and then the Qualitative Themes with quantitative data integrated within (as per a mixed methods approach). This section requires extensive restructuring to display as a mixed methods study. As it stands, the qualitative and quantitative findings do not appear integrated.
24. Please provide context to what constitutes “inner regional” and whether this is operationalized as rural.
25. Line 248, the theme “appropriateness of the program” is very unclear in the context. Would adaptation of the program elements be a better (and clearer) theme?
26. In addition, the themes need more description. For example, “patient complexity” as a stand alone theme isn’t as clear as “patient complexity influences success of virtual care”
27. Line 274, please add whether all patients received the app and what it consists of in the methods.

**DISCUSSION**

28. Lines 393-394, access was not measured explicitly. Please revise.
29. Lines 428-433, long sentence that needs restructuring for clarity.

**CONCLUSION**

30. Lines 464, again careful wording and pronouncement that this study addressed access when access/equity were not measured (unclear whether qualitative interviews addressed these questions)
